# Prevalence of Functional Limitation in COVID-19 Recovered Patients Using the Post COVID-19 Functional Status Scale

**DOI:** 10.31729/jnma.5980

**Published:** 2021-01-31

**Authors:** Pankaj Pant, Aishana Joshi, Babin Basnet, Bibek Man Shrestha, Navindra Raj Bista, Niraj Bam, Santa Kumar Das

**Affiliations:** 1 Department of Pulmonology and Critical Care, Tribhuvan University Teaching Hospital, Maharajgunj, Kathmandu, Nepal; 2 Department of General Practice and Emergency Medicine, Tribhuvan University Teaching Hospital, Maharajgunj, Kathmandu, Nepal; 3 Maharajgunj Medical Campus; 4 Department of Anaesthesiology, Tribhuvan University Teaching Hospital, Institute of Medicine, Maharajgunj, Kathmandu, Nepal

**Keywords:** *COVID-19*, *functional status*, *PCFS scale*

## Abstract

**Introduction:**

COVID-19 is an emerging global health pandemic causing tremendous morbidity and mortality worldwide. Chronic symptoms progressing to poor functional status have been reported in a substantial proportion of COVID-19 patients worldwide. This study aimed to determine the prevalence of functional limitation in COVID-19 recovered patients using the post-COVID-19 functional status scale.

**Methods:**

A descriptive cross-sectional study was conducted at Tribhuvan University Teaching Hospital. COVID-19 recovered patients with reverse transcription-polymerase chain reaction negative status were included and assessed using the post-COVID-19 functional status scale. Data entry and analysis was done in Statistical Package for the Social Sciences version 20.0. Descriptive statistics were performed.

**Results:**

A total of 106 patients were included for the final analysis. More than half of the patients (56.6%) reported having no functional limitation (grade 0), while the prevalence of some degree of functional limitation was observed in 46 (43.4%) patients (grade 1 to 4).

**Conclusions:**

Some form of functional limitation should be anticipated after COVID-19 infection. Post-COVID-19 functional status scale can be a valuable tool in determining the prevalence of functional limitation in COVID-19 recovered patients in acute health care settings. It can potentially guide in planning rehabilitative measures in post-acute care management of COVID-19 survivors.

## INTRODUCTION

Coronavirus disease 2019 (COVID-19) is caused by severe acute respiratory syndrome coronavirus-2 (SARS-COV-2). The first outbreak of SARS-COV2 was reported in Wuhan, Hubei Province in China.^1^ It was declared a global pandemic by the World Health Organization (WHO).^2^ The first confirmed case of COVID-19 from Nepal was reported in January 2020. ^3^

A wide range of pulmonary and extra-pulmonary manifestations have been reported in post-COVID-19 patients.^4–6^ Persisting symptoms with subsequent progression to poor functional status have been reported in a substantial proportion of these patients. Acute respiratory distress syndrome (ARDS), prolonged hospitalization, and admission in an Intensive Care Unit (ICU) have been reported among COVID-19 infections in acute care settings.^7,8^ The physical and mental health assessment in COVID-19 survivors with emphasis on post-acute care has been a recent global health concern.

This study aims to determine the prevalence of functional limitation in COVID-19 recovered patients using the post COVID-19 functional status (PCFS) scale.^9^

## METHODS

This is a descriptive cross-sectional study conducted in the department of Pulmonology and Critical Care at Tribhuvan University Teaching Hospital (TUTH) in December 2019. Ethical approval was taken from Institutional Review Committee, TUTH (Reference no. 152/(6-11)/E2/077/078 dated December 8, 2020). The sample size was calculated as follows:

Samples size = n
Confidence level (CI) = 95%,
For 95% CI, Z-score (Z) = 1.96
Level of significance = 5%
Sampling error (e) = 10%
Prevalence for maximum sample size (p) = 50%

$$\begin{array}{ll} \text{n} & ={\text{Z}}^{\text{2}}\text{pq}/{\text{e}}^{\text{2}}\\ & ={\left(1.96\right)}^{\text{2}}\times 0.5\times (1-0.5)/{(\text{0.1)}}^{\text{2}}\\ & =96.04\\ & \approx 96 \end{array}$$

Taking 10% non-response rate in the calculated sample size = 9.6.

Hence, minimum sample size = 96.04 + 9.6 = 105.64 ≈ 106

A convenient sampling technique was used. COVID-19 survivors with RT-PCR negative status were included in the study. Patients with active COVID-19 infection, known cardiac and/or respiratory co-morbidities with Medical Research Council (MRC) grade >0 and New York Heart Association (NYHA) grade >1 were excluded from the study. Patients with known functional disabilities and known illness likely to progress and limit physical ability like trauma, malignancies, progressive myopathies, and neuropathies were excluded from the study.

A total of 106 patients were included for the final analysis. The functional limitations were observed using the post-COVID-19 functional status (PCFS) scale (Table 1).^9^

**Table 1:** The Post COVID-19 functional status scale (PCFS)^9^.

How much are you currently affected in your everyday life by COVID-19 (Please indicate which one of the following statements applies to the most)	Corresponding PCFS scale Grade	Interpretation
I have no limitations in my everyday life and no symptoms, pain, depression or anxiety related to the infection.	0	No functional limitations
I have negligible limitations in my everyday life as I can perform all usual duties/activities, although I still have persistent symptoms, pain, depression or anxiety.	1	Negligible functional limitations
I suffer from limitations in my everyday life as I occasionally need to avoid or reduce usual duties/activities or need to spread these over time due to symptoms, pain, depression or anxiety. I am, however, able to perform all activities without any assistance.	2	Slight functional limitations
I suffer from limitations in my everyday life as I am not able to perform all usual duties/activities due to symptoms, pain, depression or anxiety. I am, however, able to take care of myself without any assistance.	3	Moderate functional limitations
I suffer from severe limitations in my everyday life: I am not able to take care of myself and therefore I am dependent on nursing care and/or assistance from another person due to symptoms, pain, depression or anxiety.	4	Severe functional limitations

Patients were graded from 0 to 4 according to the PCFS scale. The patients falling in all grades except grade 0 were observed to have some degree of functional limitation, which was further graded from 1 to 4 into negligible, slight, moderate, and severe groups depending on the severity of functional limitation (Table 1). Data were obtained using a semi-structured questionnaire self-reported by the patients. Data entry and analysis was done in SPSS version 20.0. Descriptive statistics were performed, and results were interpreted in frequency and percentage.

## RESULTS

A total of 106 patients with RT-PCR negative status were included for final analysis. The mean age of patients was 38.48±16.20 years, with a range from 22 to 83 years. The highest number of patients was 15-29 years in both sexes (Table 1).

**Figure 1:**
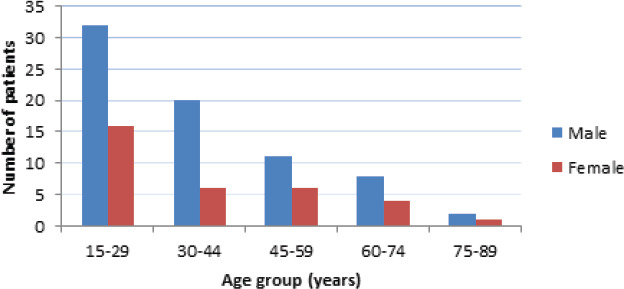
Age distribution of the patients

There were 73 (68.9%) males and 33 (31.1%) females. Nearly half of the patients (47.2%) were health care workers who comprised doctors (26.4%), nurses (12.3%), and paramedical staff (8.5%). Hypertension was the most common co-morbidity seen in 20 (18.9%) patients, followed by Diabetes mellitus in 15 (14.2%) patients. Seventeen percent of these patients required hospital admission during COVID-19 infection (Table 1).

**Table 2:** Baseline characteristics of the patients (n = 106).

Characteristics	n (%)
Sex	
Male	73 (68.9)
Female	33 (31.1)
Residence	
Kathmandu valley	67 (63.2)
Outside Kathmandu valley	39 (36.8)
Health care workers	
Yes	50 (47.2)
No	56 (52.2)
Occupation	
Doctors	28 (26.4)
Nurses	13 (12.3)
Paramedics	9 (8.5)
Students	15 (14.2)
Business	6 (5.7)
Service	19 (17.9)
Dependent	7 (6.6)
Others	9 (8.5)
Cigarette smoking	
Current smoker	12 (11.3)
Past smoker	23 (21.7)
Never smoked	71 (67)
Co-morbidities	
None	66 (62.3)
Hypertension	20 (18.9)
Diabetes mellitus	15 (14.2)
Post Tuberculosis (TB) sequelae	8 (7.5)
COPD	6 (5.7)
Thyroid disorder	5 (4.7)
Asthma	3 (2.8)
Vaccination	
BCG	85 (80.2)
Pneumococcal (within last 5 years)	5 (4.7)
Influenza (within last 1 year)	14 (13.2)
Need for hospitalization	
Yes	18 (17)
No	88 (83)

**Table 3:** Grading of patients according to the Post COVID-19 Functional Status (PCFS) scale (n = 106).

Post COVID-19 functional status scale grade	Interpretation	n (%)
Grade 0	No functional limitations	60 (56.6)
Grade 1	Negligible functional limitations	29 (27.3)
Grade 2	Slight functional limitations	13 (12.3)
Grade 3	Moderate functional limitations	2 (1.9)
Grade 4	Severe functional limitations	2 (1.9)

During the post-COVID-19 recovery state after RT-PCR negative status, more than half of the patients (56.6%) reported having no functional limitation (PCFS grade 0), while the prevalence of some degree of functional limitation was observed in 46 (43.4%) patients (Table 3).

The majority of patients (89.6%) had at least one of the pulmonary or extra-pulmonary symptoms during COVID-19 infection. A minority (11%) did not report any symptoms. Fever was the most predominant symptom observed in two-third of the patients (n=70, 66%) followed by cough in 59 (55.7%) and fatigue in 48 (45.3%) patients (Table 4).

**Table 4:** Predominant symptoms during COVID-19 infection (n = 106).

Predominant symptoms during COVID-19 infection	n (%)
Asymptomatic	11 (10.4)
Fever	70 (66)
Cough	59 (55.7)
Fatigue	48 (45.3)
Loss of smell	45 (42.5)
Loss of taste	40 (37.7)
Shortness of breath	23 (21.7)
Sore throat	15 (14.2)
Chest pain	12 (11.3)
Myalgia	10 (9.4)
Headache	5 (4.7)
Loose stool	4 (3.8)
Abdominal pain	3 (2.8)
Vomiting	2 (1.9)

## DISCUSSION

We assessed 106 COVID-19 recovered patients with RT-PCR negative status using the post-COVID-19 functional status (PCFS) scale. PCFS scale is a simple tool developed recently by Klok FA et al.^9^ to monitor the course of symptoms and its impact on patients' functional status in COVID-19 survivors. PCFS scale covers the full spectrum of functional outcomes and focuses on both limitations in usual duties/activities and lifestyle changes in five scale grades ranging from 0 to 4. Based on the PCFS scale, we observed that during the post-COVID-19 recovery state after RT-PCR negative status, more than half of the patients (56.6%) reported having no functional limitation (PCFS grade 0) while the prevalence of some degree of functional limitation was observed in 46 (43.4%) patients. Nearly one fourth (27.3%) of the patients had negligible functional limitation (PCFS grade 1). Slight functional limitation (PCFS grade 2) was seen in 13 (12.3%) patients. Moderate (PCFS grade 3) and severe functional limitations (PCFS grade 4) were observed in a minority of patients with an equal proportion of patients (n=2, 1. 9%) in each of these groups. We observed that more than 50% of our patients had no functional limitation with corresponding PCFS grade 0. This could have been partly due to the inclusion of a higher number of younger patients in our study. The mean age of patients in our study was 38.48±16.20 years, with the highest number of patients in the age group of 15-29 years in both sexes. Younger patients are reported to have a better outcome as compared to the frail elderly population after COVID-19 infection.^10–12^ Strict isolation protocols, as adapted widely across many countries in the management of COVID-19 patients in hospitals, patients' own residence or at COVID isolation centers, have resulted in a significant reduction of patient's mobility.^13^ This could be specifically implicated for the decline in functional status in COVID-19 patients during recovery state.

Nearly 90% of patients reported at least one of the clinical symptoms. Fever was the most predominant symptom observed in two-third of the patients (66%) followed by cough (55.7%). Shortness of breath and chest pain were other pulmonary symptoms observed in 21.7% and 11.3% of patients, respectively. Persisting symptoms with subsequent progression to poor functional status have been reported in a substantial proportion of COVID-19 survivors.^14,15^ Pulmonary function impairment^16^, mental health problems,^17^ and reduced quality of life^18^ to a various extent have been reported in COVID-19 patients. These factors could have a long term impact on physical, mental, social and cognitive health and well being of COVID-19 infected patients, causing a decline in functional status.

The long term consequences of COVID-19 may vary extensively among patients. In view of the massive number of COVID-19 survivors who require long-term follow-up, a simple, easy, reproducible, and cost-effective tool would be vital for proper utilization of resources in post-acute care COVID-19 patients and guide rehabilitative measures. Klok FA et al.^9^ have recommended using the PCFS scale depending on local conditions in which it is implemented. We used this tool in our study as it is simple and inexpensive and hence, could be specifically beneficial in resource-poor health care settings. We recommend large multi-centre studies with a greater sample size to validate the PCFS scale as a tool for assessing long-term post-COVID-19 functional status in the Nepalese population.

## CONCLUSIONS

The physical and mental health effects of SARS-COV2 infection should be anticipated in every COVID-19 patient. Early detection of functional decline with subsequent planning of rehabilitative measures is vital in post-acute care management of COVID-19 patients. PCFS scale is a simple tool in determining the prevalence of functional limitation in COVID-19 recovered patients in acute health care settings. We recommend large multi-centre studies with a longer duration of follow-up to validate the PCFS scale to assess long-term health effects and post COVID-19 functional status in the Nepalese population.
